# Antimicrobial activity of *Weissella cibaria* strains and the contribution of organic acids against food-associated target microorganisms

**DOI:** 10.1007/s42770-026-02029-0

**Published:** 2026-07-29

**Authors:** Camila Gonçalves Teixeira, Cleonice Aparecida Salgado, Tomás Gomes Reis Veloso, Sidney Rodrigues de Jesus Silva, Cecilia de Souza Costa, Solimar Gonçalves Machado, Cinzia Lucia Randazzo, Antônio Fernandes de Carvalho

**Affiliations:** 1 InovaLeite - Laboratório de Pesquisa em Leites e Derivados, Department of Food Technology, Universidade Federal de Viçosa, Viçosa, MG 36570-900 Brazil; 2 Department of Agriculture, Food and Environment (Di3A), University of Catania, Via S. Sofia 100, Catania, 95123 Italy; 3 ProBioEtna SRL, Spin Off at the University of Catania, Via S. Sofia 100, Catania, 95123 Italy

**Keywords:** Lactic acid bacteria, Organic acids, Antimicrobial activity, Bioprotection, Dairy products

## Abstract

*Weissella cibaria*, a heterofermentative lactic acid bacterium, is recognized for its antimicrobial activity against a range of pathogenic microorganisms. Therefore, the objective of this study was to evaluate the antimicrobial potential of* W. cibaria* strains, with an emphasis on their organic acids, against target microorganisms commonly associated with milk and dairy products (*Listeria monocytogenes*, *Escherichia coli, Staphylococcus aureus*, and *Salmonella*). To this end, this study evaluated the minimum inhibitory concentration (MIC) of cell-free supernatants (CFS) from three *W. cibaria strains* (W21, W25, and W42) and their 1:1:1 mixture. Furthermore, the CFS mixture was used in a cheese-simulating matrix to inhibit the target microorganisms. Using HPLC, the organic acids present in the CFS were identified and quantified. Finally, combinations of identified synthetic organic acids were tested for effective inhibition of the target microorganisms. The CFS from strains W21 and W25 and the CFS mixture exhibited better MIC, while a higher concentration of the CFS from W42 was required to inhibit the tested strains. In a cheese-simulating matrix, the CFS mixture significantly reduced the survival rate of the target microorganisms (p < 0.05). By HPLC, the organic acids detected were lactic, acetic, isovaleric, propionic, butyric, valeric, and malic acids. Strain W42 stood out for the absence of isovaleric and valeric acids, while strain W25 stood out for the absence of malic acid. A total of 127 combinations of synthetic organic acids were formulated. *L. monocytogenes* and *Salmonella* were predominantly inhibited by combinations of three acids, while *E. coli* and *S. aureus* were predominantly inhibited by combinations of 4 or 5 acids and 3 or 5 acids, respectively. The predominance of lactic, acetic, and isovaleric acids was associated with inhibition of the tested microorganisms, while valeric acid was not required for the inhibitory effect of the most effective combinations. These findings support the use of *W. cibaria* CFS as a potential natural antimicrobial strategy in dairy products and indicate that organic acid combinations may contribute to the inhibition of food-associated microorganisms.

**Supplementary Information:**

The online version contains supplementary material available at 10.1007/s42770-026-02029-0.

## Introduction

Milk is a biologically essential secretion from the mammary glands of healthy, well‑nourished animals, comprising a complex emulsion of lipids, proteins, carbohydrates, amino acids, vitamins and minerals, which makes it one of the most nutritionally complete animal‑derived foods [1, 2]. Despite its nutritional value, milk has physicochemical characteristics, such as high-water activity, neutral pH and rich nutrient content, which predispose it to contamination by spoilage and pathogenic microorganisms and may jeopardize sensory and technological quality, industrial yields and public health. Contamination can occur at multiple points along the production chain, exacerbated by inadequate milking, handling, storage and transport practices [3, 4].

Foodborne pathogens of major concern in milk and dairy products include *Salmonella*, *Escherichia coli* and *L. monocytogenes*, which have been implicated in outbreaks of gastroenteritis, septicemia and listeriosis associated with raw milk, contaminated cheeses and other foods [5–9]. Bovine mastitis, frequently caused by *Staphylococcus aureus*, is an additional source of contamination and zoonotic risk; *S. aureus* enterotoxins can induce severe foodborne illness and impair product safety and shelf life [10–12].

Given the microbiological hazards and consumer demand for “clean‑label” solutions, protective bacterial cultures that acidify the matrix represent a promising intervention. Organic acids exert antimicrobial effects via non‑dissociated molecules that diffuse across microbial membranes and dissociate intracellularly, lowering cytoplasmic pH, inhibiting glycolysis, reducing ATP synthesis and impairing active transport [13–15]. Acetic, citric, lactic and malic acids are GRAS compounds widely used in food preservation and can also affect biofilm formation and quorum sensing [16].

*Weissella cibaria* is recognized for exhibiting inhibitory activity against various foodborne pathogens, reducing their survival and colonization capacity in food matrices and, consequently, contributing to the reduction in the use of chemical preservatives. This antimicrobial activity has been attributed to the production of various metabolites derived from bacterial metabolism, such as organic acids (mainly lactic and acetic acids), bacteriocins, hydrogen peroxide, and other antimicrobial compounds [15, 17–20]. However, in most studies, the inhibitory effect is often generally attributed to the set of metabolites produced by the strains, while the specific contributions of individual organic acids, as well as their possible combinations, remain poorly understood.

In this context, this study aimed to evaluate the antimicrobial potential of cell-free supernatants from three autochthonous strains of *W. cibaria* (W21, W25, and W42) against *L. monocytogenes*, *E. coli*, *S. aureus*, and *Salmonella* Typhimurium, as well as to elucidate the contribution of organic acids produced by *W. cibaria*, applied individually and in combination, against these target microorganisms.

## Materials and methods

### Microorganisms and culture conditions

The strains *Weissella cibaria* W21, *W. cibaria* W25 were isolated from pastures and *W. cibaria* W42 was isolated from soil in Campos das Vertentes, Minas Gerais, Brazil [21]. Four reference strains were used as target microorganisms: *L. monocytogenes* ATCC 15313, *Escherichia coli* ATCC 11229, *Staphylococcus aureus* subsp. *aureus* ATCC 6538 and *Salmonella enterica* subsp. *enterica* serovar Typhimurium ATCC 14028. Before each experiment, the *W. cibaria* strains were pre-cultured overnight in Man, Rogosa and Sharpe broth (MRS, Oxoid Ltd., Basingstoke, United Kingdom) at 30 °C, while each target microorganism was pre-cultured separately overnight in Brain Heart Infusion broth (BHI, Oxoid Ltd.) at 37 °C.

### Determination of minimum inhibitory concentration (MIC) *in vitro*

The minimum inhibitory concentration (MIC) of compounds produced by *W.* cibaria was determined in BHI broth. *W. cibaria* (W21, W25 and W42) were grown in MRS broth at 30 °C for 24 h. After growth, the cultures were centrifuged at 10,000 × *g* for 10 min at 4 °C and the supernatant was then filtered with 0.45 μm pore membranes (Millex^®^, Merck Millipore), to obtain the cell-free supernatant (CFS). In addition, a combined CFS sample was prepared by mixing equal volumes (1:1:1) of CFS from *W. cibaria* strains. Different concentrations of *W. cibaria* CFS were evaluated, ranging from 5% to 50% (v/v), with 5% increments.

The MIC in BHI broth was determined in 96-well microplates, as described by Teixeira et al. [21]. Dilutions were prepared directly on the plates by completing each well with BHI broth to a final volume of 200 µL. The target strains were previously grown separately in BHI broth at 37 °C for 24 h and were then inoculated into the microplates at a final concentration of 1 × 10^4^ CFU/mL. The microplates were incubated at 37 °C for 24 h in a microplate reader (Multiskan™ GO, Thermo Fisher Scientific Inc., USA). Antimicrobial activity was assessed by reading the optical density at a wavelength of 600 nm (OD₆₀₀). The MIC was defined as the lowest concentration of CFS that completely inhibited growth of the target strain, indicated by the absence of OD₆₀₀ growth.

### *In situ* antimicrobial activity

 In situ antimicrobial screening was performed according to the model proposed by Garnier et al. [22], with adaptations. The CFS of the three strains of *W. cibaria* were mixed in an equal ratio (1:1:1). To simulate the cheese matrix, standardized ultrafiltered milk retentate prepared based on Hannon et al. [23], with modifications, was used. Raw milk was heated to 50 °C, skimmed in a centrifuge (Westfalia, Cuijk, Netherlands), and microfiltered (Membralox tubular membrane, 1.4 μm pore) at 50 °C (WGM, Brazil). The retentate was then ultrafiltered (0.02 μm) at 50 °C, added with sterile sodium chloride (0.7% w/v), standardized to 2% fat (w/v), and heated at 95 °C for 2 min. This preparation was added to 6-well plates, along with different concentrations of the CFS mixture, ranging from 20% to 50% (v/v), for a final volume of 10 mL. Before inoculation, each target microorganism was grown separately in BHI broth at 37 °C for 24 h and adjusted to obtain a final concentration of 1.0 × 10⁴ CFU/mL per well, followed by homogenization. The rennet (Maxiren 180; DSM) was diluted according to the manufacturer’s instructions, filtered (0.22 μm; Kasvi), and incorporated into the matrix. After incubation for 24 h at 37 °C, the microbial count was determined and expressed in Log10 (CFU/g). The control was prepared under the same experimental conditions, without the addition of the CFS mixture.

### Identification and quantification of organic acids

To identify and quantify the organic acids produced by *W. cibaria*, the CFS of each strain was treated according to the method described by Siegfried et al. [24]. Organic acids were detected and quantified using a high-performance liquid chromatograph (HPLC). The mobile phase used was 0.005 M H_2_SO_4_; the flow rate was 0.7 mL/min. The injection volume was 10 µL, and the column temperature was maintained at 45 °C. Organic acids were identified and quantified using external standard curves for lactic, acetic, propionic, butyric, valeric, isovaleric, and malic acids (Sigma, St. Louis, MO). MRS broth, processed under the same conditions as the CFS, was used as a control.

### Antagonistic activity of synthetic organic acid mixtures

Synthetic organic acid solutions were prepared from individual organic acids of analytical grade (≥ 99% purity): acetic, lactic, propionic, valeric, isovaleric, butyric, and malic acids, obtained from Sigma-Aldrich (Merck KGaA, Darmstadt, Germany). The proportions of each acid in the mixtures were defined based on the quantitative composition previously determined by HPLC in the CFS from the *W. cibaria* strains analyzed. The acid solutions were tested individually and in combinations to compare the inhibitory effect of acid-only formulations with the activity observed for the complete CFS, which may also contain other bioactive metabolites. The efficacy of the synthetic combinations against the target microorganisms was assessed through a frequency analysis of acid occurrence in the most effective mixtures allowing the identification of acids showing the highest recurrence and consistency among treatments exhibiting inhibitory activity.

### Statistical analyses

Statistical analyses were performed using GraphPad Prism software (version 8.4.3; GraphPad Software, San Diego, CA, USA). Means were compared between groups by two-way analysis of variance (ANOVA) followed by Tukey’s multiple comparisons test with a significance level of *p* < 0.05.

## Results and discussion

### Determination of MIC

**Fig. 1:**
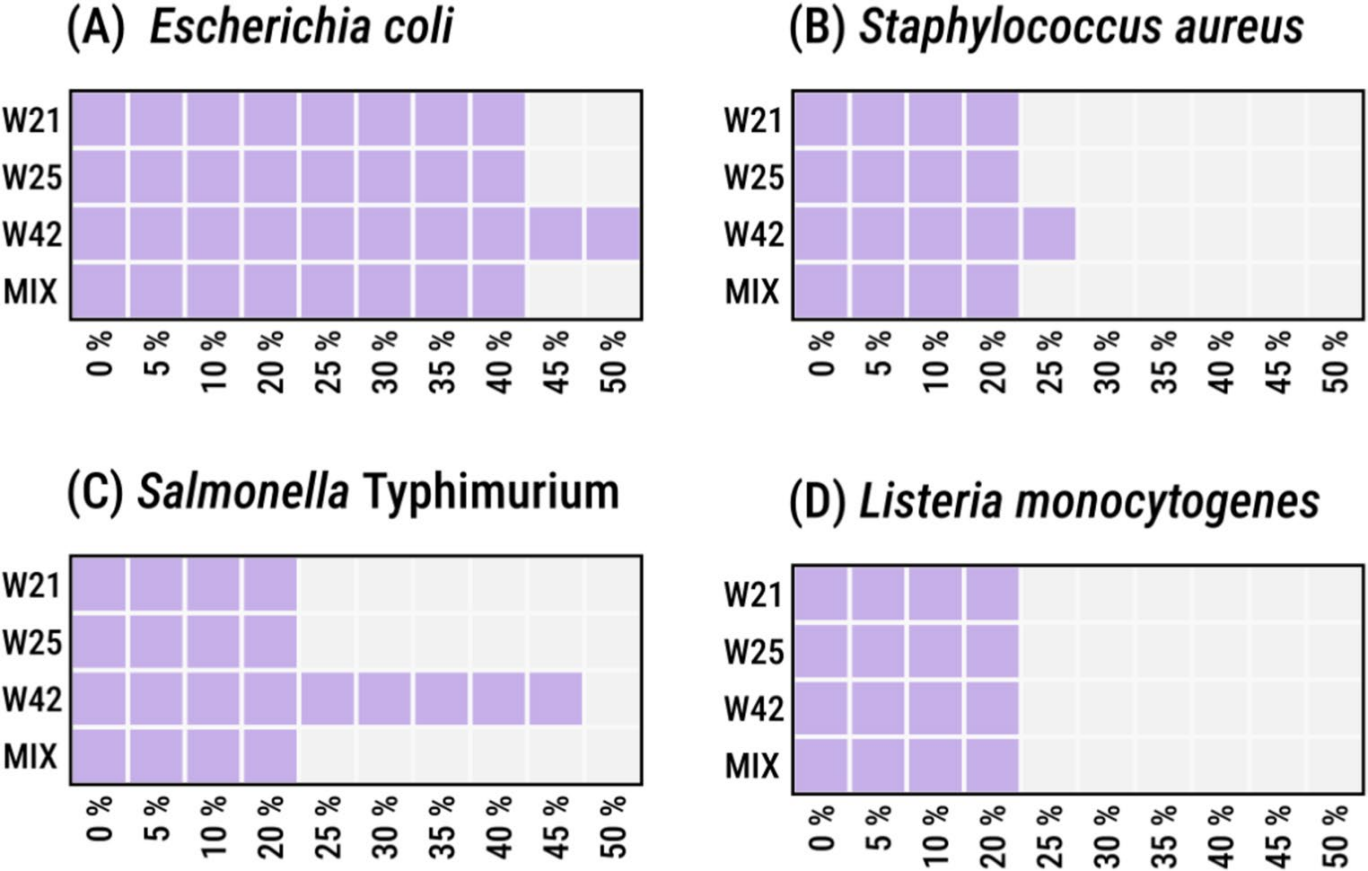
Heatmap of the minimum inhibitory concentration (MIC) in BHI (Brain Heart Infusion) broth of the cell-free supernatant of the *Weissella cibaria* W21, W25, and W42 strains, and of the mixture of these three strains in an equal ratio (1:1:1), against the following target microorganisms: (**A**) *Escherichia coli*, (**B**) *Staphylococcus aureus*, (**C**) *Salmonella* Typhimurium, and (**D**) *Listeria monocytogenes*. The colors represent the presence (lilac) or absence (gray) of microbial growth

Evaluation of the MIC of the CFS of *W. cibaria* strains revealed antimicrobial activity against the tested target microorganisms (Fig. 1). The results indicated that a 25% concentration of the CFS of strains W21, W25, as well as the mixture of the CFS from three strains (1:1:1 ratio), was sufficient to inhibit the growth of *S. aureus*. For strain W42, a 30% concentration was required (Fig. 1B). In the case of *E. coli*, greater resistance was observed, requiring concentrations of 45% of the CFS of W21, W25, and the mixture to achieve inhibition, while the CFS of strain W42 required a concentration greater than 50% (Fig. 1A). For *S.* Typhimurium, inhibition was obtained with 25% of the CFS of W21, W25, and the mixture, and 50% for the CFS of strain W42 (Fig. 1C). *L. monocytogenes* showed greater sensitivity, being inhibited by all CFS tested at a concentration of 25% (Fig. 1D). These results demonstrate variability in the antimicrobial efficacy among W. cibaria strains and indicate that the combined CFS maintained similar inhibitory activity to that of the individual supernatants.

Previous studies have shown the antimicrobial efficacy of *W. cibaria* derived metabolites against target microorganisms, due to the production of a range of antimicrobial substances. Lim et al. (2018) characterized the supernatant of *W. cibaria* and identified secreted proteins with antibacterial properties, which function to degrade the bacterial cell wall. Im et al. [25] revealed that the supernatant of *Weissella koreensis* possesses antibacterial activity and that this activity is due to organic acids, including lactic, acetic, phosphoric, succinic, pyroglutamic, citric, malic, and formic acids. Yeu et al. [26] showed that the CFS of *W. cibaria* exhibits antibiofilm activity against respiratory pathogens such as *Streptococcus pyogenes*, and *S. aureus*, resulting in a reduction in biofilm formation of up to 93%. Therefore, the antimicrobial activity of metabolites produced by *W. cibaria* strains is a desirable characteristic for food bioprotection applications, highlighting the potential of *W. cibaria* as promising biopreservatives due to their production of organic acids, bacteriocins, and several other inhibitory metabolites.

### *In situ* antimicrobial activity

**Fig. 2:**
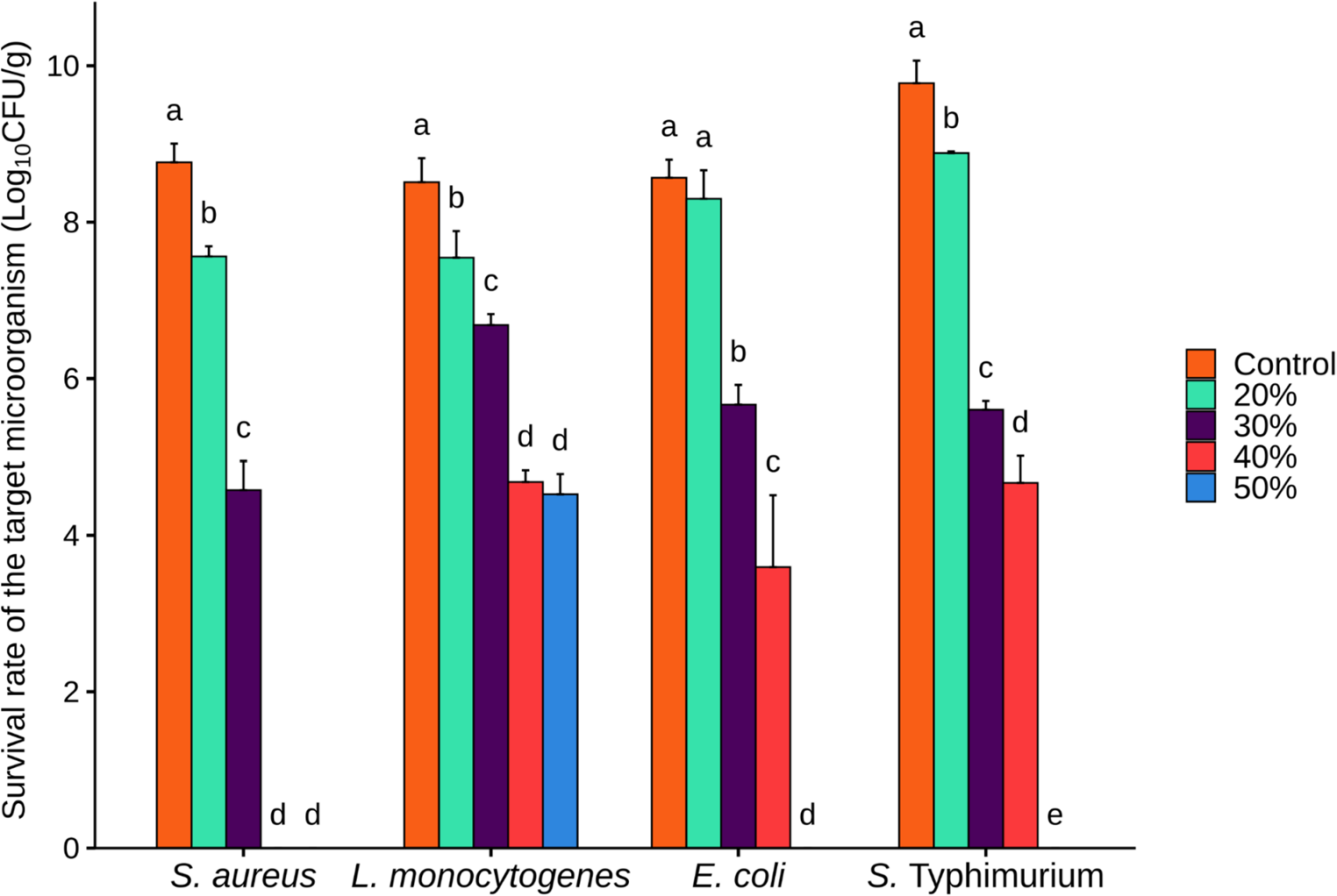
**S**urvival rate of target microorganisms after treatment with a cell-free supernatant mixture of *Weissella cibaria* strains in a cheese-simulating matrix. Surviving populations of target microorganisms are presented on a logarithmic scale. Different letters indicate statistically significant differences between samples for the same target microorganism (*p* < 0.05; ANOVA followed by Tukey’s test)

The in situ study was performed using the CFS mixture rather than the individual CFS, because the CFS mixture of *W. cibaria* strains was effective in inhibiting the target strains in vitro. The antimicrobial activity of the CFS mixture of *W. cibaria* strains against four target microorganisms was evaluated in a cheese-mimicking matrix. All treatments with the CFS mixture showed a significant reduction compared to the control (*p* < 0.05), except for the treatment with 20% of the mixture for *E. coli*, in which there was no statistically significant difference (*p* > 0.05) (Fig. 2). This in situ result reinforces the greater resistance of *E. coli* observed in the in vitro approach. Furthermore, the in situ assays showed the need for a higher concentration of CFS due to the composition of the matrix, which contains proteins and fats that can reduce the efficacy observed in BHI broth. Other studies also show that higher concentrations of antimicrobials are required in food systems to inhibit microorganisms than in growth media [27–29].

The results demonstrate that the antimicrobial efficacy of the supernatants is significantly dose-dependent and effective against Gram-positive and Gram-negative bacteria. This efficacy can be attributed to the presence of the organic acids, as well as of bacteriocins and other bioactive metabolites produced by *W. cibaria* strains [26, 30, 31].

These findings show that the CFS mixture has promising potential as a bioprotective agent in a cheese-mimicking matrix. Nevertheless, because the CFS is a complex mixture, the observed inhibition cannot be attributed exclusively to organic acids. Therefore, the next step was to determine whether synthetic organic acids, formulated according to the profile detected in the CFS, could reproduce part of the inhibitory activity against the target microorganisms.

### Identification and quantification of organic acids produced by *Weissella cibaria* strains

**Fig. 3:**
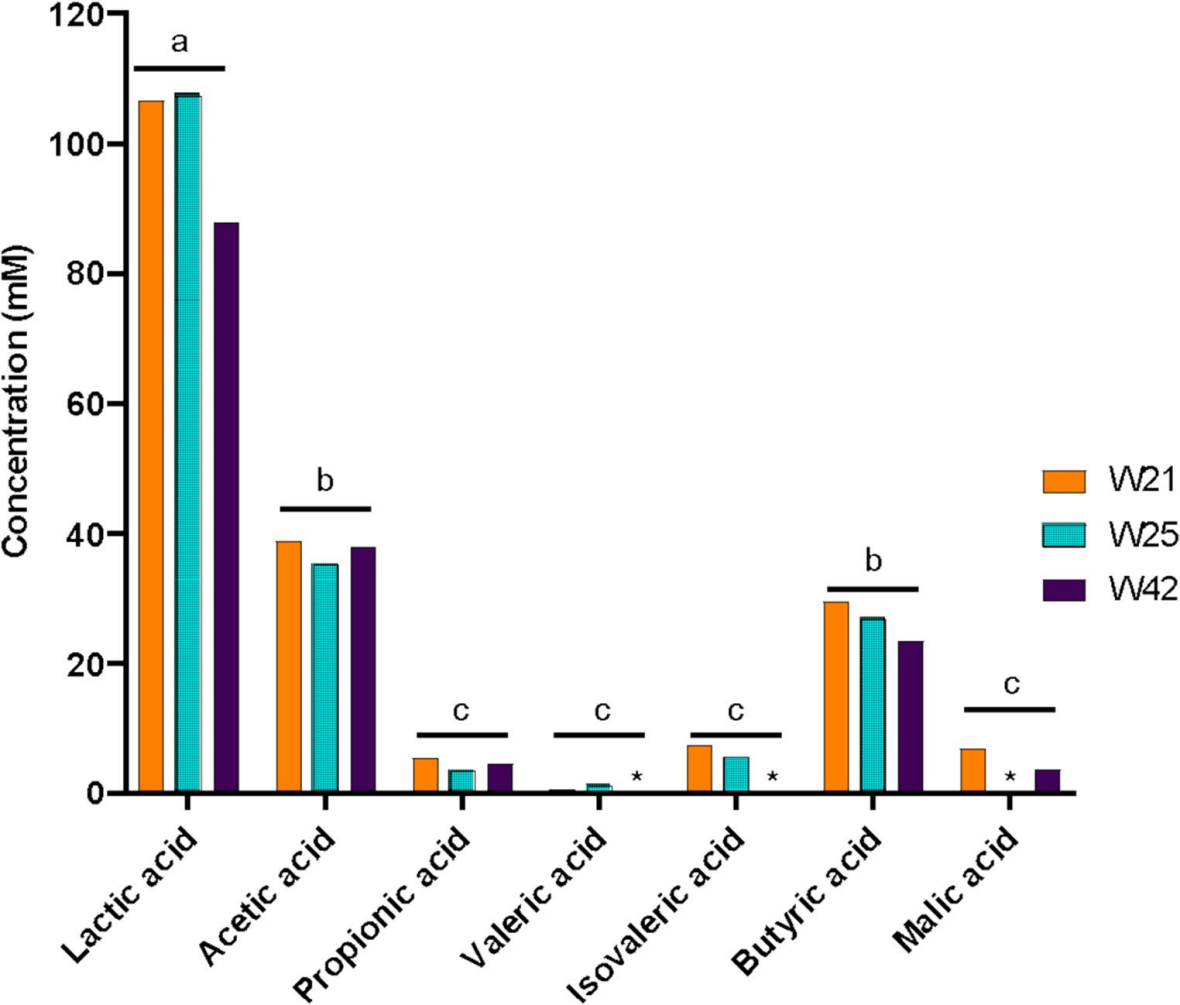
Concentration of organic acids present in the cell-free supernatant of strains of *Weissella cibaria*, determined by high-performance liquid chromatography. No statistically significant differences were observed between samples for the same acid (*p* > 0.05). However, significant differences were found between acid types (*p* < 0.05). Different letters indicate statistical differences between acids within the sample set (W21, W25, and W42). * organic acid not detected

In order to assess whether the organic acids produced by *W. cibaria* contribute to the inhibition of the target microorganisms, their identification and quantification were performed by HPLC. The identification of organic acids present in the CFS of *W. cibaria* strains W21, W25, and W42 revealed distinct metabolic profiles. The acids identified were: lactic, acetic, propionic, isovaleric, valeric, malic, and butyric acids. The CFS of strain W42 stood out for the absence of valeric and isovaleric acids, while the CFS of strain W25 was the only one in which malic acid was not identified (Fig. 3). For the same identified and quantified acid, there were no statistically significant differences between the CFS samples of W21, W25, and W42 (*p* > 0.05). Among the quantified organic acids, lactic acid presented the highest concentration, measuring 106.7 mM for W21, 107.5 mM for W25, and 87.9 mM for W42, with a statistically significant difference in relation to the others (*p* < 0.05) (Fig. 3). Then, intermediate concentrations of acetic and butyric acids were observed, which did not differ significantly from each other (*p* > 0.05) (Fig. 3). Propionic, valeric, isovaleric, and malic acids presented the lowest concentrations and also did not differ significantly from each other (*p* > 0.05) (Fig. 3). It is worth mentioning that the absence of valeric and isovaleric acids in the CFS of strain W42 may be related to the lower inhibition of the target microorganisms (Fig. 1).

The elevated lactic acid concentration aligns with the metabolic pathways typical of heterofermentative lactic acid bacteria, including *Weissella* spp [32, 33]. Shokryazdan et al. [34] reported that, in *Lactobacillus* strains, lactic acid constitutes the main organic acid produced, followed by acetic acid; the findings of the present study corroborate this result.

### Inhibition of target microorganisms by synthetic organic acids

**Fig. 4:**
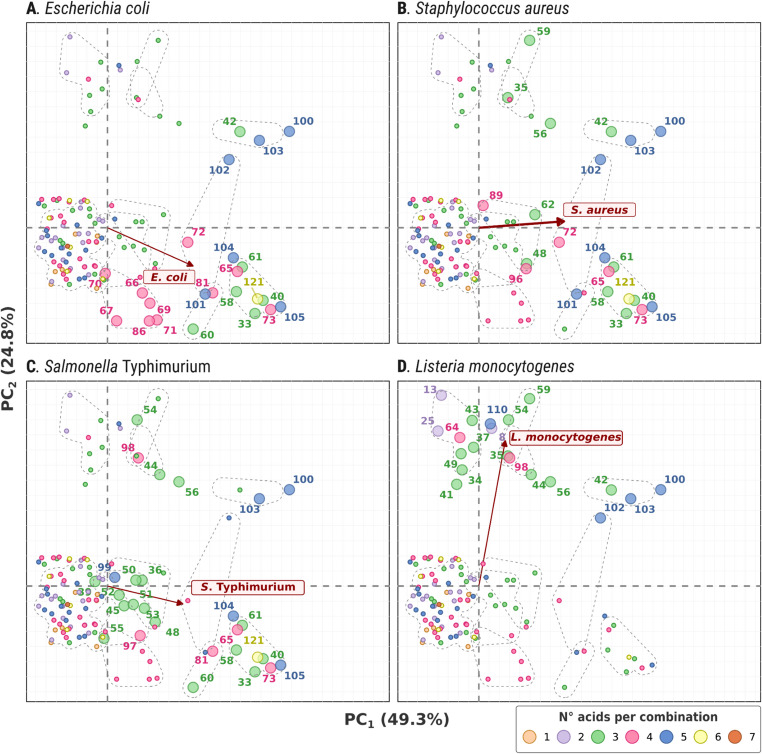
Ordination analyses based on the combination of seven organic acids in inhibiting the growth of four target microorganisms (**A**) *Escherichia coli*, (**B**) *Staphylococcus aureus*, (**C**) *Salmonella* Typhimurium, (**D**) *Listeria monocytogenes*). According to the colors of the circles, it is possible to identify the number of acids in each combination. For example, the blue circle represents a combination of 5 acids. The large and small circles represent, respectively, acid combinations that inhibit and do not inhibit target microorganism growth. Values in parentheses indicate the variation explained by each axis. Dashed lines show the groups detected by the k-means algorithm. The red arrows indicate the direction in the plot in which the combinations tend to show greater inhibitory activity against the target microorganism. Each combination is identified by a number, which can be found in Supplementary Table 1

Acids were applied both individually and in different combinations to evaluate their inhibitory effects on the target microorganisms (Fig. 4). The concentrations used for each acid corresponded to those determined in the CFS of strain W21, which presented the widest spectrum of detected acids: lactic acid (106.7 mM), acetic acid (38.8 mM), butyric acid (29.4 mM), isovaleric acid (7.5 mM), malic acid (6.9 mM), propionic acid (5.6 mM), and valeric acid (0.6 mM). In total there were 127 acid combinations (Supplementary Table 1).

**Fig. 5:**
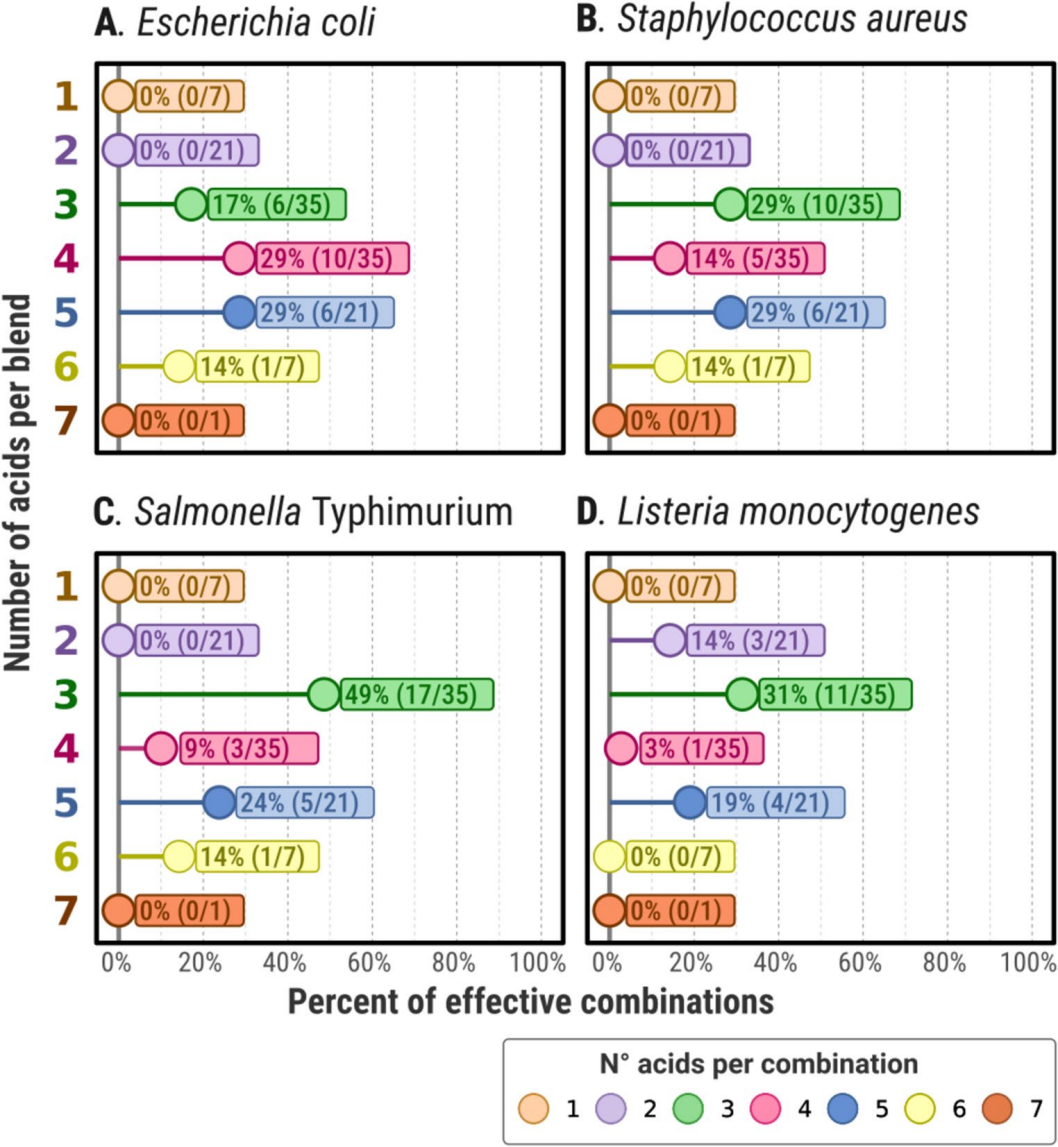
Percent inhibition of target microorganisms by each number of acid combinations. For combinations containing 1, 2, 3, 4, 5, 6, and 7 acids, it is possible to obtain, respectively, 7, 21, 35, 35, 21, 7, and 1 possible combinations. Therefore, the number of combinations that inhibited the target microorganism, in relation to the total number of possible combinations, is shown in parentheses

**Fig. 6:**
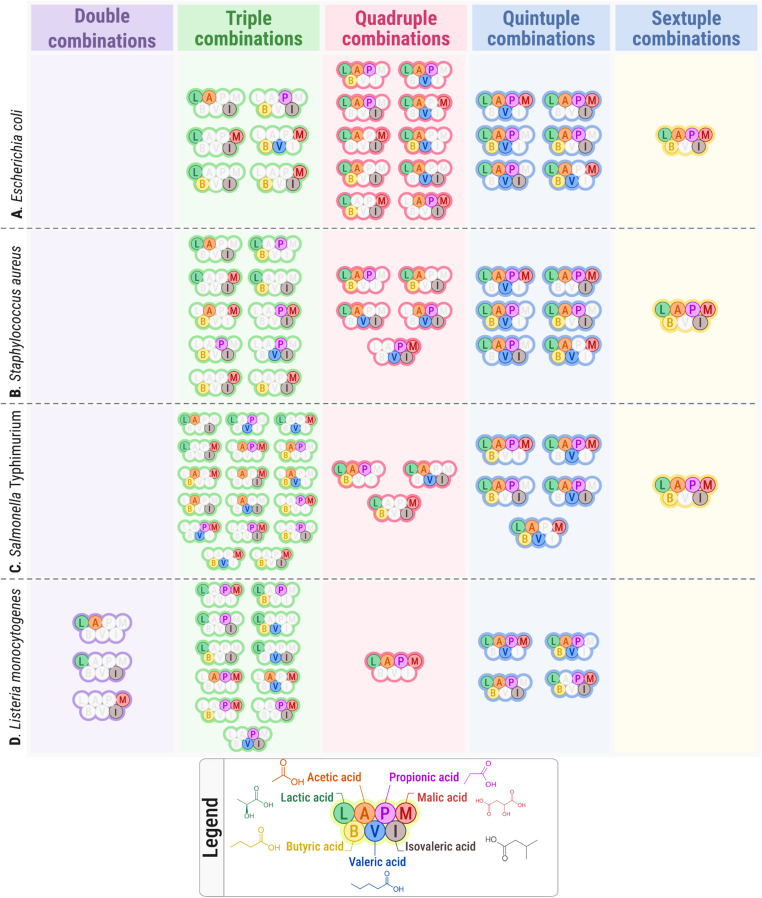
Summary of effective organic-acid combinations that inhibit target microorganisms (**A**) *Escherichia coli*, (**B**) *Staphylococcus aureus*, (**C**) *Salmonella* Typhimurium, and (**D**) *Listeria monocytogenes*). Each column displays combinations of increasing size (double, triple, quadruple, quintuple, and sextuple), while each row corresponds to one target microorganism. Acids are coded as follows: L (lactic acid), A (acetic acid), P (propionic acid), M (malic acid), B (butyric acid), V (valeric acid), and I (isovaleric acid). Colored circles show the presence of the acid in the combination, while gray combinations display absence. For example, in the “double combinations” column, only *L*. *monocytogenes* was inhibited by two-acid mixtures, specifically LA, LI, and MI; no two-acid mixtures inhibited the other target microorganisms

For *E. coli* growth inhibition, 23 effective combinations were selected (Figs. 4A and 5A). Most effective combinations involved 4 and 5 acids, followed by 3 and 6 acids, indicating that *E. coli* requires more complex mixtures to ensure total inhibition, than simpler combinations (Fig. 5A). Considering these combinations, lactic acid appears 19 times, followed by acetic acid (17), isovaleric acid (15), butyric acid (12), malic acid (11), propionic acid (11) and valeric acid (9) (Fig. 6A). This suggests that *E. coli* is more sensitive when classical fermentation acids (lactic and acetic) are present, with reinforcement of isovaleric acid.

For *S. aureus* inhibition, 22 combinations were considered (Figs. 4B and 5B). Most of the effective combinations involved 3 and 5 acids (Fig. 5B). Isovaleric acid appeared 16 times, followed by lactic acid (14), acetic, and propionic acids (13 each), butyric acid (12), malic acid (10), and valeric acid (8), indicating that these simpler combinations, mainly containing isovaleric acid, play a key role in *S. aureus* inhibition (Fig. 6B).

To inhibit *Salmonella*, 26 combinations were fully effective (Figs. 4C and 5C). Combinations with 3 acids were predominant against *Salmonella*, suggesting that simple mixtures may also be sufficient, and it is not always necessary to use more acids (Fig. 5C), with acetic acid appearing in 16 combinations, malic acid (15), butyric acid (14), isovaleric, propionic and lactic acids (13), and valeric acid (10) (Fig. 6C).

For *L. monocytogenes*, 19 combinations of synthetic organic acids completely inhibited its growth (Figs. 4D and 5D). Unlike other tested strains, *L. monocytogenes* was inhibited by double combinations. Furthermore, triple combinations prevailed, followed by quintuple and quadruple combinations (Figs. 5D and 6D). In the combinations, lactic acid prevailed (13), followed by propionic acid (12), malic and isovaleric acids (9), acetic and butyric acids (7) and valeric acid (5) (Fig. 6D).

Interestingly, of the seven possible sextuple combinations (Fig. 5), combination 121 (lactic acid, acetic acid, propionic acid, malic acid, butyric acid, and isovaleric acid) was the only one capable of inhibiting *E. coli*, *S. aureus*, and *Salmonella* (Fig. 4A, B, and C). This sextuple combination does not contain valeric acid, suggesting that valeric acid was not required for the inhibitory effect of this combination.

Acetic, lactic, and isovaleric acids were most frequently associated with inhibition of the tested strains. This result may be associated with the lower efficacy observed in the CFS of strain W42, in which isovaleric acid was not detected by HPLC (Fig. 1). This pattern indicates that short-chain acids are important for inhibiting bacterial growth, corroborating studies demonstrating their deleterious effects on cell membrane integrity, culminating in bacterial lysis [35]. Notably, branched-chain acids, such as isovaleric acid, have greater lipophilicity, enhancing their interaction with the plasma membrane, promoting ion extravasation, and resulting in reduced cell viability [35, 36].

These results indicate that inhibition of tested strains is associated with interactions between organic acids, rather than the isolated effect of a single compound. This pattern suggests that antimicrobial activity results from the combined action of organic acids, in which acidification of the medium, destabilization of the cell membrane, and interference with microbial metabolism may act in a complementary manner to enhance inhibition [37]. According to Ji et al. [38], interactions between different acids, such as hydroxycarboxylic acids (e.g., malic acid) and short-chain acids (e.g., acetic and butyric acids), can enhance antimicrobial activity.

Some in vitro studies have addressed this issue and investigated the antibacterial effect of organic acids from LAB against pathogens and spoilage organisms. Corsetti et al. [39] reported the inhibitory effects of a LAB isolate from fermented dough on fungi. They found that the organic acids acetic acid, caproic acid, formic acid, propionic acid, butyric acid, and valeric acid, tested individually at the concentrations produced by *Fructilactobacillus sanfranciscensis*, did not inhibit *Fusarium graminearum* 623, but the combination of these acids resulted in strong inhibition of the fungus. Kanjan et al. [40] demonstrated that *W. cibaria* KY10 exhibited strong antimicrobial potential against *Vibrio parahaemolyticus* T.11, mainly due to the production of organic acids such as propionic, acetic, succinic, and palmitic acids.

From an applied perspective, these findings have relevant implications for the food industry. Considering the demonstrated antimicrobial potential of *W. cibaria*, the use of CFS or formulated organic-acid combinations in cheese production appears promising as a complementary bioprotective strategy. The acid-only assays performed in this study support the contribution of organic acids to inhibition, whereas the activity of the complete CFS may also involve other bioactive metabolites produced by W. cibaria, such as bacteriocin-like substances, hydrogen peroxide, or extracellular proteins [26, 30, 31].

Regarding safety and industrial acceptance, the use of CFS or acid-based formulations may be more feasible than the direct addition of live W. cibaria cultures, because it reduces concerns related to the persistence of viable non-starter strains in the final product. Nevertheless, if live W. cibaria strains are intended for direct use as protective cultures, safety must be demonstrated at the strain level. In this regard, W. cibaria W21, W25, and W42 were previously assessed by phenotypic and genomic approaches, with no acquired antimicrobial resistance genes or mobile genetic elements detected, no known virulence factors, and no hemolytic, cytotoxic, or inflammatory effects reported [41]. Even so, broader safety assessments remain important for Weissella spp. because this genus is still less established industrially than traditional starter LAB, and strain-specific evaluation is recommended [20, 33].

The higher concentrations required in the cheese-mimicking matrix also indicate that matrix composition can reduce antimicrobial efficacy and that direct transfer of in vitro results to dairy systems should be done cautiously. In addition, short-chain and branched-chain organic acids may influence sensory attributes, especially aroma and flavor. Therefore, future studies should include challenge tests in real cheese systems, dose optimization, and sensory analysis to define concentrations that balance antimicrobial efficacy with product acceptability.

## Conclusion

This study highlights *W. cibaria* as a source of natural antimicrobial metabolites with potential application in the bioprotection of dairy products. The characterization of the organic acids presents in the cell-free supernatants revealed that lactic and acetic acids constitute an important basis of antimicrobial activity, while compounds such as isovaleric, butyric, and malic acids may contribute to broadening the spectrum and intensity of inhibition. These findings indicate that the rational formulation of acid mixtures, adjusted to the metabolic profile of *W. cibaria*, can result in antimicrobial strategies that may help reduce the use of conventional preservatives. However, the present design demonstrates combined inhibitory effects but does not formally distinguish more-than-additive from additive interactions. To enable industrial application, it is essential to ensure compliance with food safety standards and consider sensory aspects, since short-chain and branched-chain acids can impact the aroma and flavor of the final product. Thus, the balance between antimicrobial efficacy, strain safety, regulatory requirements, dose optimization, and sensory acceptability is a key element for the advancement of innovative strategies in food bioprotection.

## Supplementary Information

Below is the link to the electronic supplementary material.

Supplementary File 1 (DOCX 20.0 KB)

## Data Availability

All data generated or analysed during this study are included in this published article and its supplementary information file.

## References

[1] Linehan K, Patangia D, Ross R, Stanton C (2024) Production, composition and nutritional properties of organic milk: A critical review. Foods 13:550. 10.3390/foods13040550 38397527 10.3390/foods13040550 PMC10887702

[2] McSweeney PLH, Fox PF (2013) Advanced dairy chemistry: Volume 1A: Proteins: Basic aspects, 4th edition. Springer US

[3] Yalew K, Pang X, Huang S, Zhang S, Yang X, Xie N, Wang Y, Lv J, Li X (2024) Recent development in detection and control of psychrotrophic bacteria in dairy production: ensuring milk quality. Foods 13:2908. 10.3390/foods13182908 39335837 10.3390/foods13182908 PMC11431268

[4] Mahunu GK, Osei-Kwarteng M, Ogwu MC, Afoakwah NA (2024) Safe food handling techniques to prevent microbial contamination. Food Safety and Quality in the Global South. Springer Nature Singapore, Singapore, p pp 427-461

[5] Popa GL, Papa MI (2021) *Salmonella* spp. infection - a continuous threat worldwide. Germs 11:88–96. 10.18683/germs.2021.1244 33898345 10.18683/germs.2021.1244 PMC8057844

[6] Cho S, Jackson CR, Frye JG (2020) The prevalence and antimicrobial resistance phenotypes of *Salmonella*, *Escherichia coli* and *Enterococcus* sp. Surf water Lett Appl Microbiol 71:3–25. 10.1111/lam.13301 10.1111/lam.13301 32304575

[7] Gomes TAT, Elias WP, Scaletsky ICA, Guth BEC, Rodrigues JF, Piazza RMF, Ferreira LCS, Martinez MB (2016) Diarrheagenic *Escherichia coli*. Brazilian J Microbiol 47:3–30. 10.1016/j.bjm.2016.10.015 10.1016/j.bjm.2016.10.015 PMC5156508 27866935

[8] Swaminathan B, Gerner-Smidt P (2007) The epidemiology of human listeriosis. Microbes Infect 9:1236–1243. 10.1016/j.micinf.2007.05.011 17720602 10.1016/j.micinf.2007.05.011

[9] Uyttendaele M, De Bock T, Zhao X (2025) Emerging and reemerging food microbial hazards. Antimicrobial Strategies in the Food System: Updates, Opportunities, Challenges. Springer Nature Switzerland, Cham, pp 3–42

[10] Haider A, Ikram M, Shahzadi I, Asif Raza M (2023) Bovine Mastitis. pp 49–80

[11] McSweeney PL, FPF (2013) Advanced dairy chemistry. Springer US, Boston, MA

[12] Zigo F, Vasil’ M, Ondrašovičová S, Výrostková J, Bujok J, Pecka-Kielb E (2021) Maintaining optimal mammary gland health and prevention of mastitis. Front Vet Sci 8. 10.3389/fvets.2021.607311 10.3389/fvets.2021.607311 PMC7927899 33681324

[13] Sorathiya KB, Melo A, Hogg MC, Pintado M (2025) Organic acids in food preservation: exploring synergies, molecular insights, and sustainable applications. Sustainability (Basel) 17:3434. 10.3390/su17083434

[14] Karpiński TM, Ożarowski M (2024) Plant organic acids as natural inhibitors of foodborne pathogens. Appl Sci (Basel) 14:6340. 10.3390/app14146340

[15] Guan Y, Cui Y, Qu X, Li B, Zhang L (2025) Post-acidification of fermented milk and its molecular regulatory mechanism. Int J Food Microbiol 426:110920. 10.1016/j.ijfoodmicro.2024.110920 39316924 10.1016/j.ijfoodmicro.2024.110920

[16] Amrutha B, Sundar K, Shetty PH (2017) Effect of organic acids on biofilm formation and quorum signaling of pathogens from fresh fruits and vegetables. Microb Pathog 111:156–162. 10.1016/j.micpath.2017.08.042 28867627 10.1016/j.micpath.2017.08.042

[17] Kariyawasam KMGMM, Jeewanthi RKC, Lee N-K, Paik H-D (2019) Characterization of cottage cheese using *Weissella cibaria* D30: Physicochemical, antioxidant, and antilisterial properties. J Dairy Sci 102:3887–3893. 10.3168/jds.2018-15360 30827567 10.3168/jds.2018-15360

[18] Ren M, Jin T, Tong J, Song D, Xie Q, Li X, Li Y, Liu K, Gao J, Liu M, Cheng J (2025) Anti-inflammatory effects of *Weissella cibaria* SDS2.1 against *Klebsiella pneumoniae*-Induced mammary gland inflammation. Animals 15:1139. 10.3390/ani15081139 40281973 10.3390/ani15081139 PMC12024108

[19] Cao G, Jia M, Zhao X, Wang L, Tu X, Wang G, Nong X, Zhang Z (2016) Different effects of *Metarhizium anisopliae* strains IMI330189 and IBC200614 on enzymes activities and hemocytes of *Locusta migratoria* L. PLoS ONE 11:330189. 10.1371/journal.pone.0155257 10.1371/journal.pone.0155257 PMC4881918 27227835

[20] Fhoula I, Boumaiza M, Tayh G, Rehaiem A, Klibi N, Ouzari I (2022) Antimicrobial activity and safety features assessment of *Weissella* spp. from environmental sources. Food Sci Nutr 10:2896–2910. 10.1002/fsn3.2885 36171785 10.1002/fsn3.2885 PMC9469857

[21] Teixeira CG, Fusieger A, Martins E, Freitas R, Vakarelova M, Nero LA, Carvalho A (2021) Biodiversity and technological features of Weissella isolates obtained from Brazilian artisanal cheese-producing regions. LWT 147:111474. 10.1016/j.lwt.2021.111474

[22] Garnier L, Salas ML, Pinon N, Wiernasz N, Pawtowski A, Coton E, Mounier J, Valence F (2018) Technical note: high-throughput method for antifungal activity screening in a cheese-mimicking model. J Dairy Sci 101:4971–4976. 10.3168/jds.2017-13518 29605322 10.3168/jds.2017-13518

[23] Hannon JA, Deutsch S-M, Madec M-N, Gassi J-Y, Chapot-Chartier M-P, Lortal S (2006) Lysis of starters in UF cheeses: Behaviour of mesophilic lactococci and thermophilic lactobacilli. Int Dairy J 16:324–334. 10.1016/j.idairyj.2005.04.003

[24] Siegfried R, SG (1984) An HPLC method for determining organic acids in silage. Landwirt 37:298–304

[25] Im H, Moon J-K, Kim W-S (2016) Antibacterial activity of supernatant obtained from *Weissella koreensis* and *Lactobacillus sakei* on the growth of pathogenic bacteria. Korean J Agricultural Sci 43:415–423. 10.7744/kjoas.20160044

[26] Yeu J-E, Lee H-G, Park G-Y, Lee J, Kang M-S (2021) Antimicrobial and antibiofilm activities of Weissella cibaria against pathogens of upper respiratory tract infections. Microorganisms 9:1181. 10.3390/microorganisms9061181 34070813 10.3390/microorganisms9061181 PMC8229644

[27] Shelef LA, Jyothi EK, Bulgarellii MA (1984) Growth of enteropathogenic and spoilage bacteria in sage-containing broth and foods. J Food Sci 49:737–740. 10.1111/j.1365-2621.1984.tb13198.x

[28] Soni KA, Nannapaneni R, Schilling MW, Jackson V (2010) Bactericidal activity of lauric arginate in milk and queso fresco cheese against *Listeria monocytogenes* cold growth. J Dairy Sci 93:4518–4525. 10.3168/jds.2010-3270 20854985 10.3168/jds.2010-3270

[29] Ma Q, Davidson PM, Zhong Q (2013) Antimicrobial properties of lauric arginate alone or in combination with essential oils in tryptic soy broth and 2% reduced fat milk. Int J Food Microbiol 166:77–84. 10.1016/j.ijfoodmicro.2013.06.017 23845430 10.1016/j.ijfoodmicro.2013.06.017

[30] Yu H-S, Lee N-K, Choi A-J, Choe J-S, Bae CH, Paik H-D (2019) Antagonistic and antioxidant effect of probiotic *Weissella cibaria* JW15. Food Sci Biotechnol 28:851–855. 10.1007/s10068-018-0519-6 31093443 10.1007/s10068-018-0519-6 PMC6484072

[31] Ahmed S, Singh S, Singh V, Roberts KD, Zaidi A, Rodriguez-Palacios A (2022) The *Weissella* genus: clinically treatable bacteria with antimicrobial/probiotic effects on inflammation and cancer. Microorganisms 10:2427. 10.3390/microorganisms10122427 36557680 10.3390/microorganisms10122427 PMC9788376

[32] Styková E, Nemcová R, Maďar M, Bujňáková D, Mucha R, Gancarčíková S, Requena Domenech F (2022) Antibiofilm activity of Weissella spp. and Bacillus coagulans isolated from equine skin against Staphylococcus aureus 12:2135. 10.3390/life12122135 10.3390/life12122135 PMC9787530 36556500

[33] Fusco V, Chieffi D, Fanelli F, Montemurro M, Rizzello CG, Franz CMAP (2023) The *Weissella* and *Periweissella* genera: up-to-date taxonomy, ecology, safety, biotechnological, and probiotic potential. Front Microbiol. 10.3389/fmicb.2023.1289937 38169702 10.3389/fmicb.2023.1289937 PMC10758620

[34] Shokryazdan P, Sieo CC, Kalavathy R, Liang JB, Alitheen NB, Faseleh Jahromi M, Ho YW (2014) Probiotic potential of Lactobacillus strains with antimicrobial activity against some human pathogenic strains. Biomed Res Int 2014:1–16. 10.1155/2014/927268 10.1155/2014/927268 PMC4106073 25105147

[35] Yoon B, Jackman J, Valle-González E, Cho N-J (2018) Antibacterial free fatty acids and monoglycerides: biological activities, experimental testing, and therapeutic applications. Int J Mol Sci 19:1114. 10.3390/ijms19041114 29642500 10.3390/ijms19041114 PMC5979495

[36] Yao D, Wang X, Ma L, Wu M, Xu L, Yu Q, Zhang L, Zheng X (2022) Impact of *Weissella cibaria* BYL4.2 and its supernatants on *Penicillium chrysogenum* metabolism. Front Microbiol. 10.3389/fmicb.2022.983613 36274712 10.3389/fmicb.2022.983613 PMC9581191

[37] Xie M, Koch EHW, van Walree CA, Sobota A, Sonnen AFP, Killian JA, Breukink E, Lorent JH (2024) Synergistic effects of oxidative and acid stress on bacterial membranes of *Escherichia coli* and *Staphylococcus simulans*. Commun Biol 7:1161. 10.1038/s42003-024-06862-7 39289481 10.1038/s42003-024-06862-7 PMC11408647

[38] Ji Q-Y, Wang W, Yan H, Qu H, Liu Y, Qian Y, Gu R (2023) The effect of different organic acids and their combination on the cell barrier and biofilm of *Escherichia coli*. Foods 12:3011. 10.3390/foods12163011 37628010 10.3390/foods12163011 PMC10453431

[39] Corsetti A, Gobbetti M, Rossi J, Damiani P (1998) Antimould activity of sourdough lactic acid bacteria: identification of a mixture of organic acids produced by *Lactobacillus sanfrancisco* CB1. Appl Microbiol Biotechnol 50:253–256. 10.1007/s002530051285 9763693 10.1007/s002530051285

[40] Kanjan P, kimtun A, Chaimongkol S, Sakpetch P (2022) Probiotic *Weissella cibaria* KY10 derived from digestive tract of healthy shrimp exhibits strong antibacterial effects against *Vibrio parahaemolyticus* causing AHPND in shrimp. Aquac Res 53:2597–2607. 10.1111/are.15777

[41] Teixeira CG, Belguesmia Y, da Silva Rodrigues R, Lucau-Danila A, Nero LA, de Carvalho AF, Drider D (2024) Assessment of safety and in situ antibacterial activity of *Weissella cibaria* strains isolated from dairy farms in Minas Gerais State, Brazil, for their food application. Braz J Microbiol 55:699–710. 10.1007/s42770-023-01244-3 38253975 10.1007/s42770-023-01244-3 PMC10920571

