# Supplementary material for: Antimicrobial activity of Weissella cibaria strains and the contribution of organic acids against food-associated target microorganisms
Source: Braz J Microbiol. 2026 Jul 29;57(1):225. doi: 10.1007/s42770-026-02029-0 (PMC13421563; doi:10.1007/s42770-026-02029-0)
Supplement: Supplementary file 1 — (DOCX 20.0 KB) [file 42770_2026_2029_MOESM1_ESM.docx]

# **Supplementary Table 1.** Combinations of organic acids tested against ood-associated target microorganisms.

| Nº | Combination |
| --- | --- |
| 1 | lactic acid |
| 2 | acetic acid |
| 3 | propionic acid |
| 4 | malic acid |
| 5 | butyric acid |
| 6 | valeric acid |
| 7 | isovaleric acid |
| 8 | lactic acid, acetic acid |
| 9 | lactic acid, propionic acid |
| 10 | lactic acid, malic acid |
| 11 | lactic acid, butyric acid |
| 12 | lactic acid, valeric acid |
| 13 | lactic acid, isovaleric acid |
| 14 | acetic acid, propionic acid |
| 15 | acetic acid, malic acid |
| 16 | acetic acid, butyric acid |
| 17 | acetic acid, valeric acid |
| 18 | acetic acid, isovaleric acid |
| 19 | propionic acid, malic acid |
| 20 | propionic acid, butyric acid |
| 21 | propionic acid, valeric acid |
| 22 | propionic acid, isovaleric acid |
| 23 | malic acid, butyric acid |
| 24 | malic acid, valeric acid |
| 25 | malic acid, isovaleric acid |
| 26 | butyric acid, valeric acid |
| 27 | butyric acid, isovaleric acid |
| 28 | valeric acid, isovaleric acid |
| 29 | lactic acid, acetic acid, propionic acid |
| 30 | lactic acid, acetic acid, malic acid |
| 31 | lactic acid, acetic acid, butyric acid |
| 32 | lactic acid, acetic acid, valeric acid |
| 33 | lactic acid, acetic acid, isovaleric acid |
| 34 | lactic acid, propionic acid, malic acid |
| 35 | lactic acid, propionic acid, butyric acid |
| 36 | lactic acid, propionic acid, valeric acid |
| 37 | lactic acid, propionic acid, isovaleric acid |
| 38 | lactic acid, malic acid, butyric acid |
| 39 | lactic acid, malic acid, valeric acid |
| 40 | lactic acid, malic acid, isovaleric acid |
| 41 | lactic acid, butyric acid, valeric acid |
| 42 | lactic acid, butyric acid, isovaleric acid |
| 43 | lactic acid, valeric acid, isovaleric acid |
| 44 | acetic acid, propionic acid, malic acid |
| 45 | acetic acid, propionic acid, butyric acid |
| 46 | acetic acid, propionic acid, valeric acid |
| 47 | acetic acid, propionic acid, isovaleric acid |
| 48 | acetic acid, malic acid, butyric acid |
| 49 | acetic acid, malic acid, valeric acid |
| 50 | acetic acid, malic acid, isovaleric acid |
| 51 | acetic acid, butyric acid, valeric acid |
| 52 | acetic acid, butyric acid, isovaleric acid |
| 53 | acetic acid, valeric acid, isovaleric acid |
| 54 | propionic acid, malic acid, butyric acid |
| 55 | propionic acid, malic acid, valeric acid |
| 56 | propionic acid, malic acid, isovaleric acid |
| 57 | propionic acid, butyric acid, valeric acid |
| 58 | propionic acid, butyric acid, isovaleric acid |
| 59 | propionic acid, valeric acid, isovaleric acid |
| 60 | malic acid, butyric acid, valeric acid |
| 61 | malic acid, butyric acid, isovaleric acid |
| 62 | malic acid, valeric acid, isovaleric acid |
| 63 | butyric acid, valeric acid, isovaleric acid |
| 64 | lactic acid, acetic acid, propionic acid, malic acid |
| 65 | lactic acid, acetic acid, propionic acid, butyric acid |
| 66 | lactic acid, acetic acid, propionic acid, valeric acid |
| 67 | lactic acid, acetic acid, propionic acid, isovaleric acid |
| 68 | lactic acid, acetic acid, malic acid, butyric acid |
| 69 | lactic acid, acetic acid, malic acid, valeric acid |
| 70 | lactic acid, acetic acid, malic acid, isovaleric acid |
| 71 | lactic acid, acetic acid, butyric acid, valeric acid |
| 72 | lactic acid, acetic acid, butyric acid, isovaleric acid |
| 73 | lactic acid, acetic acid, valeric acid, isovaleric acid |
| 74 | lactic acid, propionic acid, malic acid, butyric acid |
| 75 | lactic acid, propionic acid, malic acid, valeric acid |
| 76 | lactic acid, propionic acid, malic acid, isovaleric acid |
| 77 | lactic acid, propionic acid, butyric acid, valeric acid |
| 78 | lactic acid, propionic acid, butyric acid, isovaleric acid |
| 79 | lactic acid, propionic acid, valeric acid, isovaleric acid |
| 80 | lactic acid, malic acid, butyric acid, valeric acid |
| 81 | lactic acid, malic acid, butyric acid, isovaleric acid |
| 82 | lactic acid, malic acid, valeric acid, isovaleric acid |
| 83 | lactic acid, butyric acid, valeric acid, isovaleric acid |
| 84 | acetic acid, propionic acid, malic acid, butyric acid |
| 85 | acetic acid, propionic acid, malic acid, valeric acid |
| 86 | acetic acid, propionic acid, malic acid, isovaleric acid |
| 87 | acetic acid, propionic acid, butyric acid, valeric acid |
| 88 | acetic acid, propionic acid, butyric acid, isovaleric acid |
| 89 | acetic acid, propionic acid, valeric acid, isovaleric acid |
| 90 | acetic acid, malic acid, butyric acid, valeric acid |
| 91 | acetic acid, malic acid, butyric acid, isovaleric acid |
| 92 | acetic acid, malic acid, valeric acid, isovaleric acid |
| 93 | acetic acid, butyric acid, valeric acid, isovaleric acid |
| 94 | propionic acid, malic acid, butyric acid, valeric acid |
| 95 | propionic acid, malic acid, butyric acid, isovaleric acid |
| 96 | propionic acid, malic acid, valeric acid, isovaleric acid |
| 97 | propionic acid, butyric acid, valeric acid, isovaleric acid |
| 98 | malic acid, butyric acid, valeric acid, isovaleric acid |
| 99 | lactic acid, acetic acid, propionic acid, malic acid, butyric acid |
| 100 | lactic acid, acetic acid, propionic acid, malic acid, valeric acid |
| 101 | lactic acid, acetic acid, propionic acid, malic acid, isovaleric acid |
| 102 | lactic acid, acetic acid, propionic acid, butyric acid, valeric acid |
| 103 | lactic acid, acetic acid, propionic acid, butyric acid, isovaleric acid |
| 104 | lactic acid, acetic acid, propionic acid, valeric acid, isovaleric acid |
| 105 | lactic acid, acetic acid, malic acid, butyric acid, valeric acid |
| 106 | lactic acid, acetic acid, malic acid, butyric acid, isovaleric acid |
| 107 | lactic acid, acetic acid, malic acid, valeric acid, isovaleric acid |
| 108 | lactic acid, acetic acid, butyric acid, valeric acid, isovaleric acid |
| 109 | lactic acid, propionic acid, malic acid, butyric acid, valeric acid |
| 110 | lactic acid, propionic acid, malic acid, butyric acid, isovaleric acid |
| 111 | lactic acid, propionic acid, malic acid, valeric acid, isovaleric acid |
| 112 | lactic acid, propionic acid, butyric acid, valeric acid, isovaleric acid |
| 113 | lactic acid, malic acid, butyric acid, valeric acid, isovaleric acid |
| 114 | acetic acid, propionic acid, malic acid, butyric acid, valeric acid |
| 115 | acetic acid, propionic acid, malic acid, butyric acid, isovaleric acid |
| 116 | acetic acid, propionic acid, malic acid, valeric acid, isovaleric acid |
| 117 | acetic acid, propionic acid, butyric acid, valeric acid, isovaleric acid |
| 118 | acetic acid, malic acid, butyric acid, valeric acid, isovaleric acid |
| 119 | propionic acid, malic acid, butyric acid, valeric acid, isovaleric acid |
| 120 | lactic acid, acetic acid, propionic acid, malic acid, butyric acid, valeric acid |
| 121 | lactic acid, acetic acid, propionic acid, malic acid, butyric acid, isovaleric acid |
| 122 | lactic acid, acetic acid, propionic acid, malic acid, valeric acid, isovaleric acid |
| 123 | lactic acid, acetic acid, propionic acid, butyric acid, valeric acid, isovaleric acid |
| 124 | lactic acid, acetic acid, malic acid, butyric acid, valeric acid, isovaleric acid |
| 125 | lactic acid, propionic acid, malic acid, butyric acid, valeric acid, isovaleric acid |
| 126 | acetic acid, propionic acid, malic acid, butyric acid, valeric acid, isovaleric acid |
| 127 | lactic acid, acetic acid, propionic acid, malic acid, butyric acid, valeric acid, isovaleric acid |
